# Pathological pseudoprogression to anti-PD-1 inhibitor in metastatic periampullary carcinoma: Case report

**DOI:** 10.1097/MD.0000000000032644

**Published:** 2023-01-27

**Authors:** Junhui Wang, Yan Wang, Xiaoling Che

**Affiliations:** a Department of Radiation Oncology, The Quzhou Affiliated Hospital of Wenzhou Medical University, Quzhou People′s Hospital, RD. Minjiang, Dist. Kecheng. Quzhou, Zhejiang, China; b Department of Medical Oncology, The Quzhou Affiliated Hospital of Wenzhou Medical University, Quzhou People’s Hospital, RD. Minjiang, Dist. Kecheng. Quzhou, Zhejiang, China.

**Keywords:** efficacy, periampullary carcinoma, programmed death 1 (PD-1), pseudoprogression, repigmentation

## Abstract

Pseudoprogression has been deemed as a rare clinical phenomenon during the treatment of immune checkpoint inhibitors in patients with advanced cancers, especially in periampullary carcinoma, however, leaving potential molecular mechanism remain unknown.

**Patient concerns:**

Regular examination after radical pancreaticoduodenectomy because of periampullary carcinoma.

**Diagnoses:**

Recurrent periampullary carcinoma with metastasis in liver.

**Interventions:**

Regimens of XELOX (oxaliplatin at a dose of 130 mg/m^2^, day 1 and oral capecitabine at a dose of 1000 mg/m^2^ twice a day, day 1–14, every 21 days), and tislelizumab at a dose of 200 mg, day 1, per 21 days, was prescribed as palliative treatment.

**Outcomes:**

Pseudoprogression and symptom of hair and mustache repigmentation were also observed, which resulted in partial response finally.

**Lessons:**

Results of the present case suggested that pseudoprogression, along with hair and mustache repigmentation, possibly caused by anti-PD-1 inhibitors, may also happen in patients with periampullary carcinoma, which should be paid attention to. The potential mechanism should be further investigated.

## 1. Introduction

Periampullary carcinoma was considered to be one of rarely common, but highly aggressive cancers, which accounted for approximately 0.2% among gastrointestinal tumors.^[1]^ Treatment strategies for advanced periampullary carcinoma was determined by histological types including intestinal subtype, pancreaticobiliary subtype, and mixed one.^[2]^ Regimens for colorectal cancer, or pancreaticobiliary cancer were recommended for patients with periampullary carcinoma according to their histological subtypes, respectively.^[3]^ However, the overall survival time for advanced or metastatic disease was still limited, without sufficient treatment strategies to cure.

Tislelizumab, an investigational, humanized IgG4 monoclonal antibody with high affinity and binding specificity for programmed death 1 (PD-1), has demonstrated preliminary antitumor activity in various cancers, including lung cancer,^[4]^ esophageal squamous cell carcinoma,^[5]^ hepatocellular carcinoma,^[6]^ and hodgkin lymphoma.^[7]^ It has been approved as front line treatment option for patients with Hodgkin lymphoma in China. However, pseudoprogression during the treatment of tislelizumab occasionally occurs, which comes to be a troublesome problem to identify in clinical practice. Although histological findings by re-biopsy would be beneficial to the identification, it is not widely accepted by patients as an invasive detection. Besides, there has been no convincing noninvasive strategy discovered so far for the identification of pseudoprogression in clinical practice.

Herein, we reported a patient diagnosed with recurrent periampullary carcinoma metastasized to liver, resulted in radiologic pseudoprogression during the treatment of tislelizumab combined with chemotherapy, which was pathologically confirmed by re-biopsy subsequently. With the continuation of immunotherapy, the patient has achieved partial response, with survival time extending. We hope the presentation of the present case would provide clinical evidence of pseudoprogression during the management of patients with periampullary carcinoma treated with anti-PD-1 inhibitors.

We presented the following case in accordance with the CARE reporting checklist.

## 2. Case presentation

**Figure 1.**
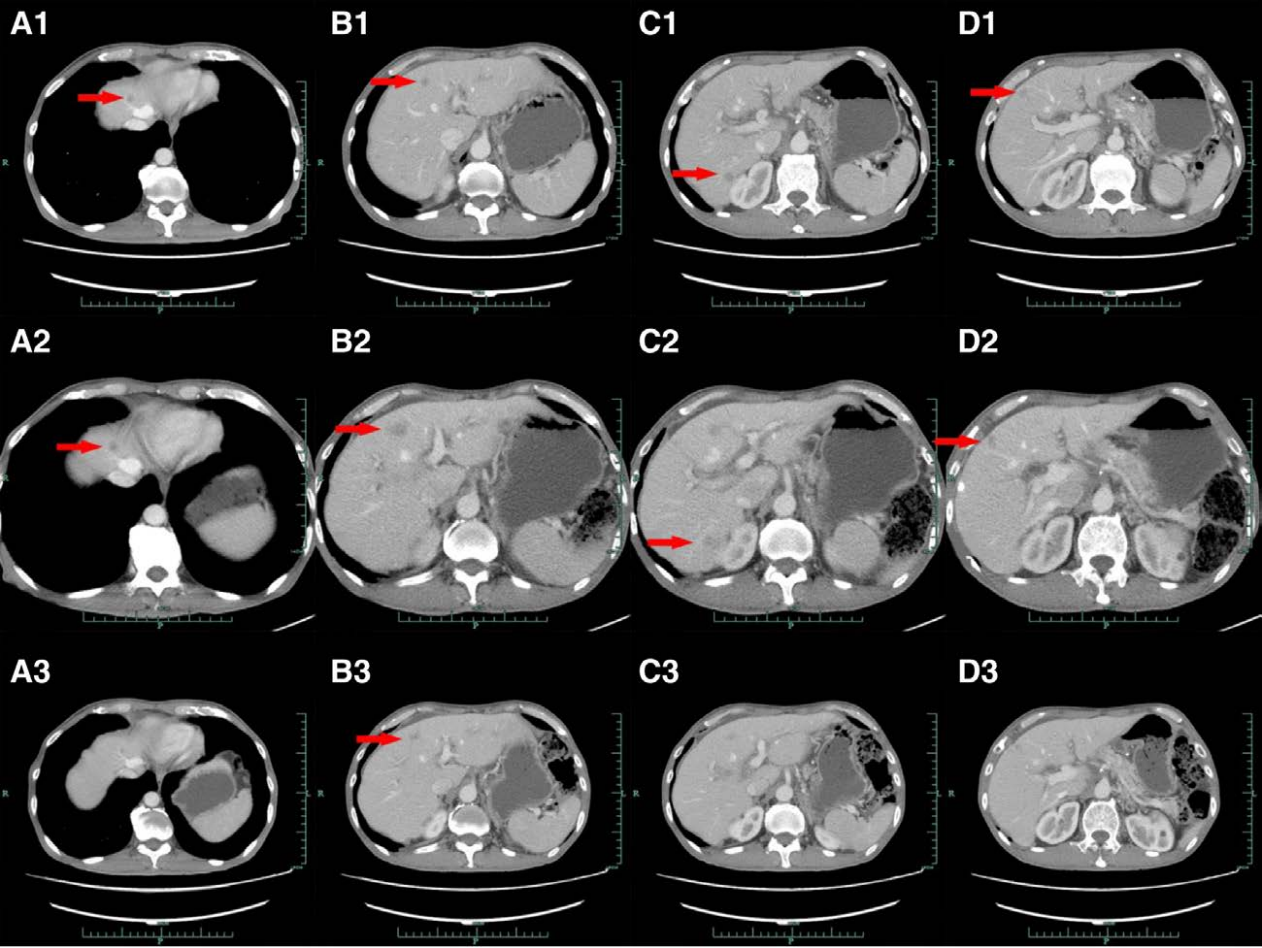
Abdomen CT scanning showed the metastatic tumors in the liver (red arrows). (A1—D1) April 24, 2020. (A2–D2) July 3rd, 2020. (A3–D3) August 17, 2020. CT = computed tomography.

**Figure 2.**
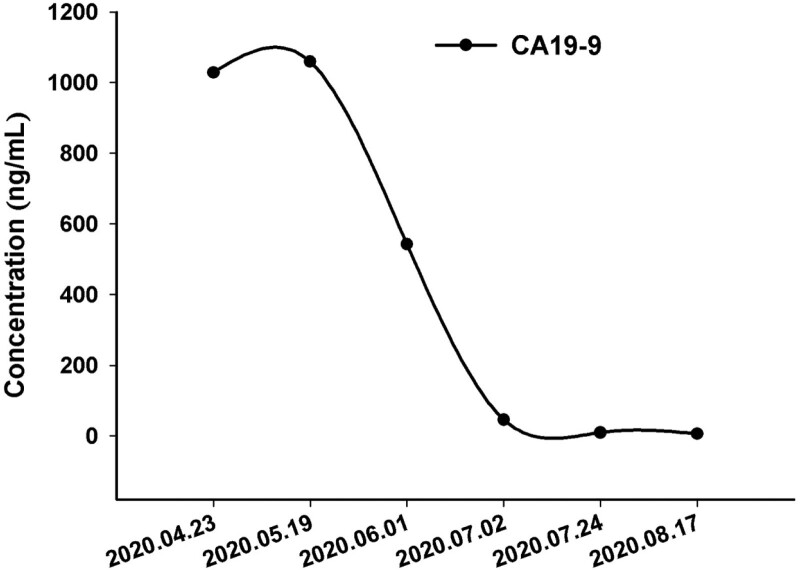
The variations of tumor marker CA19-9 during the whole treatment.

**Figure 3.**
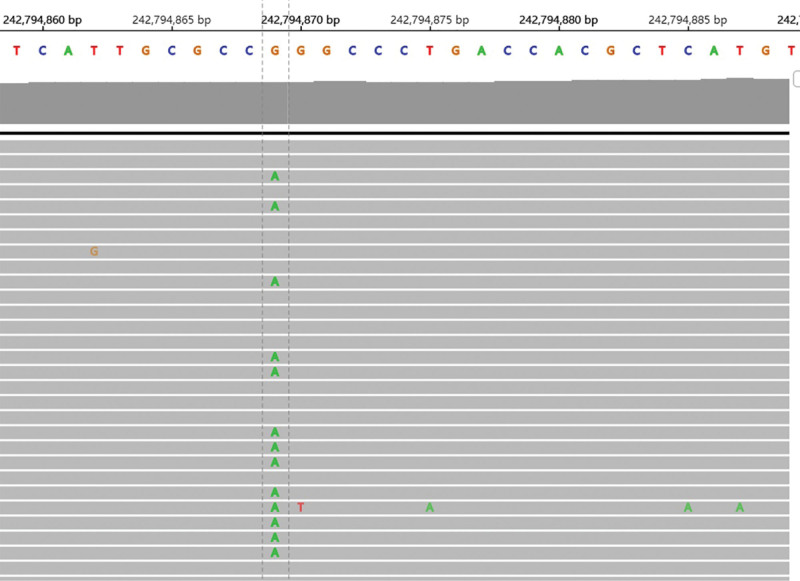
Integrative genomics viewer (IGV) screenshots displaying the chimeric reads from targeted sequencing, PD-1 exon 2 mutation (p.R114W, c.340C > T, frequency as 48.5%). PD-1 = programmed death 1.

**Figure 4.**
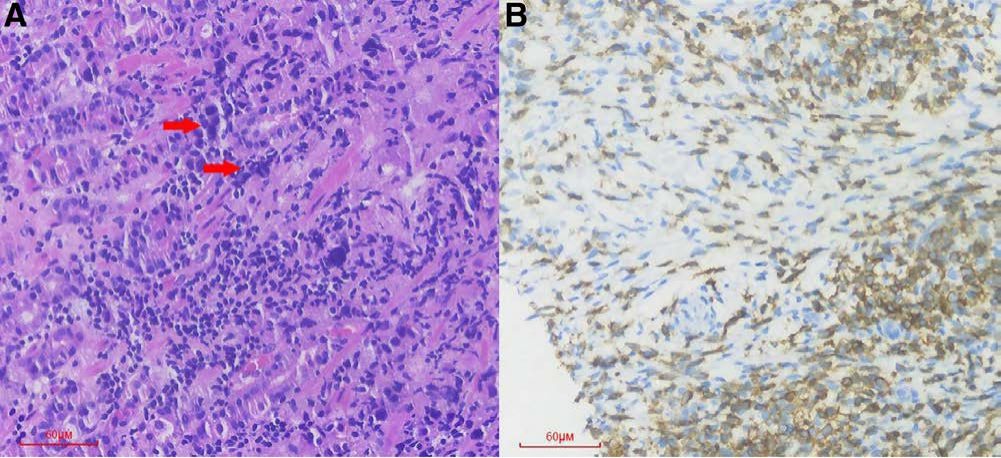
Histological findings with hematoxylin and eosin-stained (A) red arrows for tumor cells, and (B) immunohistochemistry finding with CD3 staining, for biopsy specimen from liver.

A 56-year-old man was admitted to our institution on April 22, 2020 for regular examination after radical pancreaticoduodenectomy and subsequent adjuvant chemotherapy because of periampullary carcinoma with early stage. He denied any family or other medical history. The patient had been diagnosed with periampullary carcinoma in December 2019, and received radical pancreaticoduodenectomy, as well as subsequent adjuvant chemotherapy in other hospital. Immunohistochemistry outcomes of surgical specimens were presented as: CK7 (positive), CDX2 (partial positive), MUC-1 (positive), MUC-2 (partial positive), and CK20 (partial positive). The adjuvant chemotherapy was developed with the regimen of gemcitabine at a dose of 1000 mg/m^2^, day 1, 8, and albumin-bound paclitaxel at a dose of 130 mg/m^2^, day 1, 8, per 21 days. At the time of the first visit in clinic in our institution, he had completed his adjuvant chemotherapy for 1 month. In addition, he denied smoking, alcohol history, any other medical or family history. Abdomen computed tomography (CT) suggested multiple emerged lesions in liver, with peripheral enhancement during venous phase (Fig. 1, A1–D1). Elevated tumor marker of CA19-9 also suggested the recurrence of periampullary carcinoma (Fig. 2). Otherwise, chest CT did not reveal any other occupations in lungs. Based on the history of disease and typical imageological characteristics, the patient was clinically diagnosed as recurrent periampullary carcinoma with metastasis in liver. Simultaneously, whole exon sequencing with next generation sequencing using plasma sample was conducted to search potential available targets. As a result, whole exon sequencing did not report any common mutations, but PD-1 exon 2 R114W mutation, with a high level frequency as 48.5% (Fig. 3). In terms to the mixed component with intestinal type and pancreaticobiliary type by immunohistochemistry findings, regimen of XELOX, including oxaliplatin at a dose of 130 mg/m^2^, day 1 and oral capecitabine at a dose of 1000 mg/m^2^ twice a day, day 1 to 14, as well as tislelizumab (an anti-PD-1 monoclonal antibody) at a dose of 200 mg, day 1, per 21 days, was prescribed as palliative treatment. After 2 cycles’ exposure, the patient was surprised to find his hair and mustache repigmentation (Supplementary Figure S1, Supplemental Digital Content, http://links.lww.com/MD/I320). Meanwhile, tumor marker of CA19-9 was also found decreasing significantly (Fig. 2). However, repeated abdomen CT revealed enlarged and emerging lesions in the liver, which suggested possible progression (Fig. 1, A2–D2). Based on the inconsistent outcomes between imagical results and serum tumor marker, we performed a re-biopsy on tumors in the liver to identify the nature of the progression. As a result, histological findings revealed very few tumor cells surrounded by numerous T cells (Fig. 4A). Subsequently, immunohistochemistry findings with CD3 stained also confirmed a mass of active T cells (Fig. 4B). Accordingly, pseudoprogression of cancer in the liver was considered, which was possibly caused by the immunotherapy agent of tislelizumab. Therefore, the regimen of XELOX combined with tislelizumab was continued for another 2 cycles. On August 17 after four cycles’ treatment, repeated abdomen CT replied shrinking and remission of lesions in the liver (Fig. 1, A3–D3), which confirmed the pseudoprogression of the imagical results. Tumor marker of CA19-9 fallen back to the normal range (Fig. 2). There was no immune-related adverse event observed during the whole treatment. The patient is receiving maintenance therapy with oral capecitabine and tislelizumab now, with progression-free survival time as 8 months, and still in extension.

## 3. Discussion and conclusions

The present case reported a patient diagnosed with recurrent periampullary carcinoma metastasized to liver, suffered radiologic pseudoprogression co-occurring hair repigmentation during the treatment of tislelizumab combined with chemotherapy. With the continuation of the immunotherapy, he has achieved a partial response, with survival time extending. To the best of our knowledge, it was the first manuscript to report pseudoprogression during anti-PD-1 treatment in patient with periampullary carcinoma.

Pseudoprogression, as one kind of unconventional types of response to immunotherapy in cancers, is described as transient progressive disease followed by a partial response.^[8]^ The rate of pseudoprogression was reported as 0.6% to 14.8% in patients receiving immunotherapy, including cytotoxic T lymphocyte antigen 4, PD-1, and PD-L1 inhibitors.^[9,10]^ However, the molecular mechanisms, or biomarkers for pseudoprogression was still uncovered. Several radiological and biological methods, such as positron emission tomography-CT, circulating-tumor deoxyribonucleic acid and radiomics were reported helpful to distinguish pseudoprogression from progression, however need further confirmation in larger cohort.^[10]^ In the present case, with the decreasing serum level of CA19-9 and hair repigmentation, the pseudoprogression was considered, and further evidence was provided by re-biopsy for the enlarged lesions in the liver. Based on that, we considered that an additional biopsy of progressive lesions might be essential for identification of pseudoprogression. However, as an invasive examination, biopsy might not be easily accepted by patients in clinical practice. Instead of that, a noninvasive medical examination is urgently needed.

In addition, in the present case, we occasionally detected a rare mutation of PD-1 exon 2 R114W alteration, with a high frequency as 48.5%. As an essential functional and targeted protein, the missense mutation of PD-1 appeared to be interesting and confusing. There were limited publications reported on potential significance of PD-1/PD-L1 gene polymorphic variations.^[11–13]^ A recent study reported that there were 3 major non-redundant heterozygous mutations, including A245T, V252M in exon 4 and T267S in exon 5 found in the feline PD-L1 gene, which might be related to the malignant degree of cancers. These missense mutations should be emerged as targets of checkpoint-blocking therapies in the future.^[11]^ In addition, an experimental study conducted by performing a computational analysis of 33 cancer cell lines, and identifying PD-L1 isoforms with exon 4 enrichment expression.^[14]^ Results of the study showed that cancer cells with overexpression of exon 4 enriched PD-L1 may generate a secreted form of PD-L1, which might play a significant role in T cell regulation, and result in the enhanced efficacy of immunotherapy.^[14]^ However, the published alterations of PD-1/PD-L1 were different from the specific mutation in the present case, which has been rarely reported before. Nevertheless, the mutation of PD-1 exon 2 R114W has not been reported or presented in the Gene Bank (https://www.genecards.org/) either. Hence, the potential pathological meaning of the mutation should be explained prudently and conservatively, which also need further investigation in basic experiment or future clinical practice. Hair repigmentation was considered to be a good response marker in patients receiving anti-PD1/PD-L1 therapy.^[15]^ The interesting phenomenon was also observed in the present case, though with the mechanism still uncovered.^[16,17]^ Even so, the effectiveness of subsequent treatment, as well as the interesting symptoms in the present patient should be followed and further reported.

There were limitations in the present case. The most obvious one was the mutation being detected with plasma, rather that tumor tissue. In fact, we got enough tumor tissue from the re-biopsy in the liver. However, after hematoxylin eosin staining and immunohistochemistry, there was no enough tumor tissue left, which came to be the limitation in the present study. In addition, few former literatures reported the specific mutation of PD-1 exon 2 R114W alteration, which may lead to the undefined function of that. It was suggested that basic experiment might still be essential to identify the mechanism of the mutation in the future.

Briefly, we presented a case diagnosed with recurrent periampullary carcinoma metastasized to liver, suffered radiologic pseudoprogression co-occurring hair repigmentation during the treatment of tislelizumab combined with chemotherapy. We considered the presentation of the case could raise the attention of the clinical phenomenon of pseudoprogression during the treatment of anti-PD-1 inhibitors in patients with periampullary carcinoma.

## Acknowledgment

The authors thank the patient for his participation and agreement to publication of the report.

## Author contributions

**Conceptualization:** Junhui Wang.

**Data curation:** Junhui Wang.

**Funding acquisition:** Junhui Wang.

**Investigation:** Yan Wang.

**Methodology:** Xiaoling Che.

**Resources:** Yan Wang.

**Supervision:** Xiaoling Che.

**Visualization:** Xiaoling Che.

**Writing – original draft:** Junhui Wang.

**Writing – review & editing:** Xiaoling Che.

## Supplementary Material
