# Supplementary material for: Pathological pseudoprogression to anti-PD-1 inhibitor in metastatic periampullary carcinoma: Case report
Source: Medicine (Baltimore). 2023 Jan 27;102(4):e32644. doi: 10.1097/MD.0000000000032644 (PMC9875975; doi:10.1097/MD.0000000000032644)
Supplement: Supplementary file 1 [file medi-102-e32644-s001.pdf]

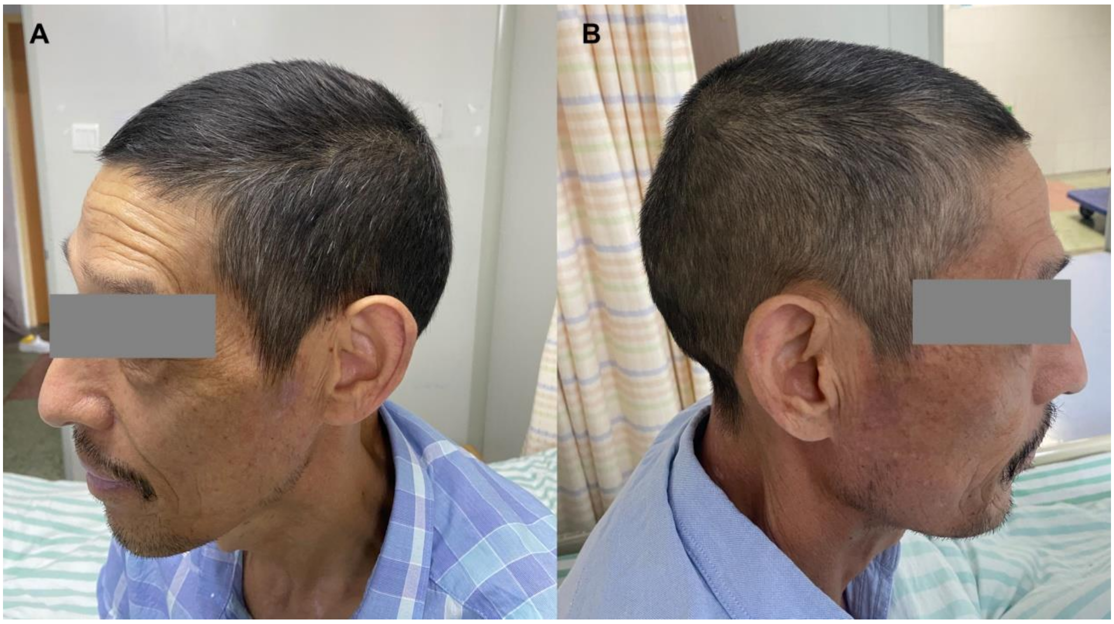

Supplemental figure. Hair and mustache repigmentation. A. Before treatment for recurrent disease. B. After two cycles of treatment.
